# Risk factors associated with drug therapy among elderly people with Alzheimer’s disease: a cross-sectional study

**DOI:** 10.1590/1516-3180.2019.0461.R2.19022020

**Published:** 2020-06-22

**Authors:** Marcela Forgerini, Maria Teresa Herdeiro, José Carlos Fernandes Galduróz, Patrícia de Carvalho Mastroianni

**Affiliations:** I Pharmacist and Doctoral Student, Department of Drugs and Medicines, School of Pharmaceutical Sciences, Universidade Estadual Paulista (UNESP), Araraquara (SP), Brazil.; II PhD. Pharmacist and Professor, Department of Medical Sciences, Universidade de Aveiro, Institute of Biomedicine (iBiMED), Aveiro, Portugal.; III PhD. Adjunct Professor, Department of Psychobiology, Universidade Federal de São Paulo (UNIFESP), São Paulo (SP), Brazil.; IV PhD. Pharmacist and Adjunct Professor, Department of Drugs and Medicines, Universidade Estadual Paulista (UNESP), Araraquara (SP), Brazil.

**Keywords:** Dementia, Patient safety, Polypharmacy, Complexity index, Medication regimen, Potentially inappropriate medication

## Abstract

**BACKGROUND:**

Improving knowledge and establishing strategies and policies for better patient safety are worldwide priorities.

**OBJECTIVE:**

To evaluate drug safety among elderly people with Alzheimer’s disease (AD).

**DESIGN AND SETTING:**

Cross-sectional study among elderly people within the National AD Assistance Protocol (PCDTDA/MS) who were living in the municipality of Araraquara, Brazil, in 2017.

**METHODS:**

Through interviews conducted with relatives/caregivers of elderly people with diagnoses of AD, the following variables were evaluated: comorbidities, drug therapy used, use of potentially inappropriate medications for the elderly (PIMs), presence of potentially inappropriate interactions (PIIs) and medication regimen complexity index. Factors associated with AD severity were also evaluated. Multivariate and simple logistic regressions were applied.

**RESULTS:**

143 elderly people enrolled in PCDTDA/MS were analyzed. The majority were women (67.1%); assisted only through the public healthcare system (75.5%); polymedicated (57.4%); using at least one PIM (63.6%); presenting at least one PII (63.6%); and under drug therapy of low to medium complexity (92.2%). No semi-annual monitoring of the effectiveness of PCDTDA/MS drugs was identified. The proportion using AD drug therapy at daily doses differing from those recommended by the World Health Organization was 75.6%. However, these doses were not associated with drug risk.

**CONCLUSION:**

The data from this study raise the hypothesis that use of polypharmacy might show a correlation with severity of AD. The drug safety risk may be associated with comorbidities of the metabolic syndrome, anxiety and off-label use of PIMs, such as risperidone and quetiapine, and benzodiazepines (i.e. clonazepam and flunitrazepam).

## INTRODUCTION

Prescription of potentially inappropriate medications for the elderly (PIMs),^1^ the complexity of drug therapy regimens,^2^ the risk of potentially inappropriate interactions (PIIs),^3^ use of polypharmacy^4^ and occurrences of adverse drug events (ADEs)^5^ are factors that compromise drug safety among elderly people.

The risk of hospitalizations due to ADE is two to seven times greater among elderly people.^6^ It has been estimated that for every two hospitalized elderly individuals, the reason for admission of one of them was possibly an occurrence of one or more ADEs.^7^

PIMs are drugs with risks that can outweigh the benefits, especially when there are safer and more effective alternatives.^8^ They are also associated with occurrences of ADEs,^9^ and their use contributes to a twofold increase in the risk of hospitalization among elderly people.^10^

In addition, some drugs that have been standardized as essential by the World Health Organization (WHO) and in the Brazilian National List of Essential Medicines (RENAME-Brazil) are considered to be PIMs. However, there is often no other safer drug alternative.^11^

With the aging of the population, there are projections of high prevalence and incidence of dementia,^12^ among which Alzheimer’s disease (AD) is the most prevalent form.^13^ However, within the context of drug therapy for these conditions, we are not aware of any study on such therapy with concomitant evaluation of drug risk factors, i.e. polypharmacy, PIM use, PII, drug therapy complexity index and comorbidities. Nor have associations of these factors with disease severity among elderly people with dementia or AD been assessed.^14^^,^^15^^,^^16^^,^^17^

## OBJECTIVE

The objectives of the present study were to characterize elderly people with a diagnosis of AD; identify comorbidities, drug therapy complexity, use of PIMs and presence of PIIs; and raise hypotheses regarding possible drug risk factors. Through this, we aimed to contribute to national and international patient safety goals.

## METHODS

### Study design and ethics

A cross-sectional study was conducted in Araraquara, Brazil, over the course of the year 2017.

The study design was based on the guidelines for Strengthening the Reporting of Observational Studies in Epidemiology (STROBE).^18^ The protocol for this study was approved by the Research Ethics Committee of the Universidade Federal de São Paulo in 2016 (no. 2.877.560).

### Setting and participants

The study was conducted at the Araraquara Reference Center for Elderly Patients (CRIA) and at the Regional Health Directorate III (DRS-III) for services of specialized nature.

Elderly individuals with a diagnosis of AD are referred by the healthcare services to the CRIA. CRIA provides medium-complexity services and specializes in geriatric care, using protocols for treating forgetfulness, dementia, stroke sequelae and mild depression.

In DRS-III, drug therapy for AD is dispensed. In 2017, 260 elderly people were registered at the DRS-III of Araraquara within the Clinical Protocol and Therapeutic Guidelines for Alzheimer’s Disease of the Ministry of Health (PCDTDA/MS),^19^ including both new and old cases.

These guidelines are documents based on scientific evidence that establish criteria for diagnosing the health problem and make recommendations for treatments and dosages and for monitoring the therapeutic results.

The elderly people who were registered within the PCDTDA/MS had been diagnosed with AD (ICD-10: G30) in accordance with the criteria of the National Institute on Aging and the Alzheimer’s Disease and Related Disorders Association, which have been endorsed by the Brazilian Academy of Neurology.^20^

AD is diagnosed through the following procedures: evaluation of the clinical history; cognitive screening through the clinical parameters of the Mini-Mental State Examination (MMSE)^21^ and the Clinical Dementia Rating (CDR);^22^ laboratory tests (vitamin B12, folic acid, electrolytes, blood glucose, urea, creatinine, alanine aminotransferase, aspartate aminotransferase and thyroid-stimulating hormone); and magnetic resonance imaging or computed tomography scans.^19^

In addition, the drug therapy that has been approved for treating AD, i.e. donepezil, galantamine and rivastigmine (acetylcholinesterase inhibitors) and memantine, is available free of charge to elderly patients who are included in the PCDTDA/MS. AD drug therapy dispensing is performed through the public healthcare system in order to ensure access and integrality of the treatment at the outpatient level.

Thus, elderly individuals who had been registered within the PCDTDA/MS were included in this study. Elderly individuals who were living in long-term institutions were excluded for ethical reasons, given the advanced stage of their disease and the absence of a relative or caregiver to participate in the interview.

### Data

This study was conducted through interviews with relatives or caregivers of elderly patients who were seen at CRIA and at the time of dispensing of AD drug therapy in DRS-III. The interview was led by one researcher.

A standardized questionnaire was drawn up for the interviews, and it sought the following information about the elderly subjects: gender; access to healthcare (only through the public healthcare system, only through the private system or through a mixture of the public and private systems); age; body mass index (BMI); schooling; monthly income; time of diagnosis of AD; family history of AD; severity of AD; clinical parameters (MMSE^21^ and CDR^22^); drug therapy and time of use; medication regimen complexity index (MRCI); defined daily dose (DDD) of AD drug therapy, stratified as above the requirement, below the requirement or adequate dose, as defined by WHO; comorbidities and time of diagnosis; and consumption of alcohol and tobacco.

The data obtained through the interview were confirmed using secondary sources, i.e. from medical records available at the healthcare service, prescriptions and clinical laboratory tests on the elderly subjects.

### Measurements

#### Comorbidities

Comorbidities were identified through the relatives’ or caregivers’ reports or through self-reports; and from secondary data sources (medical records).

#### Severity of Alzheimer’s disease

The severity of Alzheimer’s disease was assessed through insertion of memantine either in association with anticholinesterases or as monotherapy. The insertion of memantine was evaluated to ensure that this was not associated with anticholinesterase intolerance, but rather with the severity of AD, in accordance with the MMSE and CDR scores that had been pre-established within the PCDTDA/MS.^19^

#### Metabolic syndrome

Metabolic syndrome (MetS) consists of a set of factors that increase the risk of coronary heart disease.^23^

The guidelines developed through the National Cholesterol Education Program for detection of MetS recommend measurement of abdominal circumference, blood glucose levels, cholesterol levels (LDL and HDL) and blood pressure.^23^ However, since it was not possible to make these measurements in this study, the following criteria were used to define MetS: BMI of 30 kg/m^2^ or greater) and use of antihypertensives antidiabetics and antidyslipidemics, as recommended in the literature.

Thus, presence of MetS was ascertained in terms of the presence of at least three of the factors described above.

#### Medication regimen complexity index (MCRI)

The complexity of drug therapy results from the multiplicity of prescribed regimen factors.

The MRCI is an open index divided into three domains: dosage form (pharmaceutical form according to route of administration), dosing frequency (number of times the drugs is given per day/week/month) and administration instructions (i.e. tablet fractionation, fasting, etc.).^24^ The more complex the dosage schedule and the process of drug use are, the higher the score assigned will be.

The minimum score is 1.5 points, which represents a single tablet or capsule taken once a day. There is no limit to the number of drugs to be analyzed.

We stratified the MRCI score as denoting low (1.5-14 points), medium (14-28 points) or high complexity (28-42 points).

The MRCI is the gold standard for assessing the complexity of drug therapy.^25^ However, it has some limitations, considering that observation of which scores correspond to higher scores shows that there is no maximum score or stratification of these scores. This was the reason why we made our own stratification for this study.

#### Potentially inappropriate medications

Prescription guides have been developed to identify any use of drugs that is considered inappropriate for elderly individuals. These are termed potentially inappropriate medications (PIMs), i.e. drugs for which the tradeoff between risk and benefit does not justify their use.

In our study, PIM assessments were made through the following prescription guides: updated Beers criteria,^26^^,^^27^ STOPP/START version 2,^28^ French consensus panel^29^ and Canadian national consensus panel,^30^ and Strand criteria.^31^

These guides report on the drugs that are considered to be PIMs, depending on the clinical condition of the elderly individual, dose, length of time prescribed and PIM-comorbidity interactions.

#### Drug risk variables

In order to evaluate drug safety among patients with AD, we defined the following variables as drug risk variables: PIM use and PII, evaluated through the updated Beers criteria,^26^^,^^27^ STOPP/START version 2,^28^ French consensus panel^29^, Canadian national consensus panel^30^ and Strand criteria;^31^ the complexity of drug therapy according to the medication regimen complexity index (MRCI);^24^ comorbidities identified through self-reports and with confirmation from medical records; and presence of polypharmacy, which was defined as use of five or more drugs for a period greater than or equal to 90 days.

### Data analysis

#### Sample size

Given that a total of 260 elderly people had been registered within the PCDTDA/MS, the sample size for a confidence level of 90% (α = 0.05) was 133 elderly people.

### Statistical analysis

The variables of interest were described in terms of their absolute and relative frequencies.

Two statistical analyses were conducted: multivariate and simple logistic regressions.

To analyze the severity of AD in relation to the presence of drug risk variables (presence of polypharmacy, high-complexity MRCI, PIM use and occurrence of PII), multivariate logistic regression was used.

From another perspective, to evaluate the influence of each variable of this study on the severity of AD and/or in the presence of one or more variables that had been defined as drug risk variables, simple logistic regression was used. The aim of this analysis was to evaluate whether there were any variables that influenced the severity of AD. For this, AD severity and the drug risk variables were considered to be the dependent variables and the others were independent variables.

Female gender, schooling ≤ 4 years and presence of high-complexity MRCI were defined as independent variables in the simple logistic regression. Individual comorbidities were analyzed, as were comorbidities grouped in accordance with the International Statistical Classification of Diseases and Related Health Problems (ICD-10).

The statistical software used was BioEstat (version 5.3).

## RESULTS

**Figure 1.**
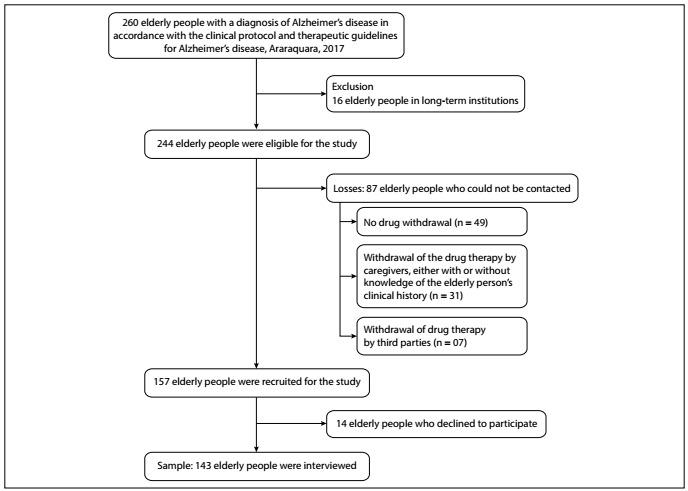
Flowchart of identification and eligibility of elderly people with Alzheimer’s disease in accordance with the clinical protocol and therapeutic guidelines for Alzheimer’s disease (PCDTDA/MS), in the municipality of Araraquara, 2017.

Out of the 260 elderly people who were enrolled in the PCDTDA/MS, 16 were excluded because they were living in long-term institutions. Thus, 244 elderly individuals were eligible for inclusion in this study. Fourteen did not agree to participate and there were another 87 losses, due to the following: AD drug therapy was not received through the public healthcare system (n = 49); AD drug therapy was dispensing to relatives or caregivers who did not know the clinical history of the elderly individual (n = 31); or AD drug therapy was dispensed through a means of transportation that had been hired just to obtain it (n = 7) (Figure 1).

The age of the elderly individuals within the PCDTDA/MS ranged from 64 to 97 years. Their median age was 81 years (Q1 = 76; Q3 = 87) and they had had their diagnosis of AD for a median period of four years (Q1 = 02; Q3 = 7.5). Most of these elderly people were women (67.1%); did not have any family history of AD (60.1%); were assisted only through the public healthcare system (75.5%); and had had less than four years of schooling (83.2%). Twenty of these elderly people were illiterate and only six of them had had higher education.

In addition, most of them were polymedicated (57.4%); were making use of at least one PIM (63.6%) and had one PII (63.6%). The mean number of drugs in use was five drugs/elderly person [standard deviation, SD: 2.69] and the drug therapy of 92.2% of these elderly individuals was classified as presenting low or medium complexity.

Furthermore, although diabetes mellitus, arterial hypertension and dyslipidemia were among the most prevalent comorbidities, most of these elderly people did not have metabolic syndrome. However, it was also noted that most of them had one or more of the prodromal symptoms of AD: anxiety, insomnia and depression. These symptoms are frequently treated with drugs that are considered to be PIMs (Table 1).

**Table 1.** Clinical conditions of the elderly people with probable diagnoses of Alzheimer’s disease (n = 143), Araraquara, 2017

Variable	n (%)
Gender	
Female	96 (67.1)
Male	47 (32.9)
Body mass index (kg/m^2^)	
Median	24.2
Quartile	(Q1 = 21.9/Q3 = 28.3)
Low-weight (< 18)	6 (4.2)
Normal (18-24)	62 (43.3)
Overweight (25-30)	36 (25.2)
Obesity (30-35)	12 (8.4)
Moderate obesity (35-40)	5 (3.5)
Severe obesity (> 40)	1 (0.7)
Without knowledge	21 (14.7)
Drugs in use (n)	
0-1 drug	8 (5.6)
2-4 drugs	53 (37.1)
5-10 drugs	76 (53.1)
> 10 drugs	6 (4.2)
Medication regimen complexity index (MRCI)	
Low	70 (48.9)
Medium	62 (43.3)
High	11 (7.80)

Comorbidities	
Mental and behavioral disorders (ICD)	
Insomnia (F51)	85 (59.4)
Anxiety (F06.4)	81 (56.6)
Depression (F32)	56 (39.2)
Depression without treatment (F32)	9 (6.3)
Schizophrenia (F2054)	2 (1.4)
Panic syndrome (F41)	1 (0.7)
Attention deficit hyperactivity disorder (F90)	1 (0.7)
Diseases of the circulatory system (ICD)	
Arterial hypertension (I10)	77 (53.8)
Stroke (I64)	17 (11.9)
Infarction (I21.9)	8 (5.6)
Angina pectoris (I20)	4 (2.8)
Arrhythmia (I49.9)	4 (2.8)
Endocrine and metabolic diseases (ICD)	
Dyslipidemia (E78)	41 (28.7)
Diabetes mellitus (E11)	35 (24.5)
Metabolic syndrome	23 16.1)
Hypothyroidism (E039)	20 (14.0)
Obesity (E66)	17 (11.9)
Deficiency of cholecalciferol (E55)	10 (7.0)
Nervous system diseases (ICD)	
Epilepsy (G40)	8 (5.6)
Parkinson’s disease (G20)	2 (1.4)
Diseases of the musculoskeletal and connective system (ICD)	
Osteoporosis (M81.9)	6 (4.2)
Gout (M10)	5 (3.5)
Osteoarthritis (M19.9)	4 (2.8)
Arthritis (M13.9)	4 (2.8)
Blood diseases and hematopoietic organs (ICD)	
Anemia (D50)	10 (7.0)
Respiratory system diseases (ICD)	
Asthma (J45)	3 (2.1)
Chronic obstructive pulmonary disease (J44.9)	1 (0.7)
Diseases of the genitourinary system (ICD)	
Benign prostatic hyperplasia (N40)	9 (6.4)
Infectious and parasitic diseases (ICD)	
Viral hepatitis C (B18.2)	1 (0.7)
Neoplasms (ICD)	
Prostate cancer (C61)	3 (2.1)
Breast cancer (C50)	2 (1.4)
Others	
Labyrinthitis (H83)	3 (2.1)
Mean number of morbidities/elderly person	4 (SD: ± 2)

ICD = International Classification of Diseases; SD = standard deviation.

Among the drugs used, which were available through PCDTDA/MS, galantamine (46.1%) and donepezil (33.61%) were the ones most prescribed. Most of the elderly individuals were receiving monotherapy (88%), at daily doses above the defined daily dose that are recommended by the WHO (60.2%) (Table 2).

**Table 2.** Description of drug therapy for Alzheimer’s disease (AD) and defined daily dose*,* stratified according to disease stage, among elderly people with a diagnosis of Alzheimer’s disease (n = 143), Araraquara, 2017

Drug therapeutic protocol (ATC)	n	Cumulative frequency [%]	DDD (mg)	Dose used/ day (mg)	n (%)
Mild stage of Alzheimer’s disease (n = 121)					
Galantamine (N06DA04)	66	46.1	16	8	8 (5.6)
				16	13 (9.0)
				24	45(31.5)
Donepezil (N06DA02)	48	79.7	7.5	5	7 (4.9)
				10	41 (28.7)
Rivastigmine (N06DA03)	7	84.6	9.0	4.5	1 (0.7)
				6.0	6 (4.2)
Moderate stage of Alzheimer’s disease (n = 16)					
Galantamine + memantine	10	95.8	-	24 + 20	10 (7.0)
Donepezil + memantine	5	99.3	-	10 + 20	5 (3.5)
Rivastigmine + memantine	1	100	-	4.5 + 20	1 (0.7)
Severe stage of Alzheimer’s disease (n = 6)					
Memantine (N06DX01)	6	88.8	20	20	6 (4.2)

ATC = Anatomical Therapeutic Chemical; DDD = defined daily dose; n = number of elderly.

It was noted that no record of MMSE and CDR screening tests has yet been identified, thus making it impossible to evaluate the effectiveness of anticholinesterase treatment. Therefore, no semiannual reevaluation of the effectiveness of these tests that had been predicted through the PCDTDA/MS was done.

Ninety-one of the elderly subjects (63.6%) used at least one drug that was considered to be a PIM. The mean number of PIMs in use was around two per elderly individual. The incidence of PII was higher among the polymedicated elderly individuals (mean of 2.3 PIIs per elderly person [SD: 1.44]) than among the non-polymedicated individuals (mean of 1.5 PIIs per elderly person [SD: 1.22]).

The most frequently used PIM classes were antipsychotics (44.6%), such as risperidone and quetiapine; and benzodiazepines (BZD) (27.3%), such as clonazepam and flunitrazepam. The most frequent PIIs were of drug-illness type. The largest proportion of PIIs came from use of BZD and Z drugs among elderly people with dementia and depression (41.2%). Additionally, use of antipsychotics without a diagnosis of psychosis (34.5%) stood out (Table 3).^26^^,^^27^^,^^28^^,^^29^^,^^30^^,^^31^

**Table 3.** Frequency of use of potentially inappropriate medications (PIMs) and occurrence of potentially inappropriate interactions (PIIs), according to the assessment instruments and rationale, among elderly people with a probable diagnosis of Alzheimer’s disease (n = 143), Araraquara, 2017

	n (%)	Rationale	Assessment instrument
Potentially inappropriate medications (ATC)			
Antipsychotics	64 (44.6)		
Quetiapine (N05AH04)	33 (23.0)	Increased risk of stroke and greater rate of cognitive decline and mortality among individuals with dementia. Potential risk of falls and fractures.	Updated Beers criteria (2015)^27^
Risperidone (N05AX08)	28 (19.6)	Use of antipsychotics for behavioral problems and delirium among patients with dementia should be avoided.	Updated Beers criteria (2015)^27^
Pericyazine (N05AC01)	3 (2.0)	Potential risk of falls and fractures.	French consensus panel^29^
Benzodiazepines	39 (27.3)^*^		
Short-to-medium half-life			
Lorazepam (N05BA06)	1 (0.7)	Elderly people have increased sensitivity to benzodiazepines and decreased metabolism of long‐acting agents.	Updated Beers criteria (2003 and 2015)^26^^,^^27^
Alprazolam (N05BA12)	4 (2.8)	In general, independent of half-life, all benzodiazepines increase the risks of cognitive impairment, delirium, falls, fractures and worsening of respiratory failure.	Updated Beers criteria (2003 and 2015)^26^^,^^27^
Estazolam (N05CD04)	1 (0.7)		Updated Beers criteria (2015);^27^ French consensus panel^29^
Long half-life			
Clonazepam (N03AE01)	18 (12.6)	The rationale for long half-life benzodiazepines is the same as for short and medium. However, this use may be appropriate for eye movement sleep disorders, benzodiazepine withdrawal, severe anxiety disorder and periprocedural anesthesia.	Updated Beers criteria (2015)^27^
Flunitrazepam (N05CD03)	9 (6.3)		French consensus panel^29^
Bromazepam (N05BA08)	4 (2.8)		French consensus panel^29^
Diazepam (N05BA01)	2 (1.4)		Updated Beers criteria (2003 and 2015)^26^^,^^27^
Nitrazepam (N05CD02)	2 (1.4)		French consensus panel^29^
Flurazepam (N05CD01)	1 (0.7)	This has an extremely long half-life in elderly patients, such that it promotes prolonged sedation.	Updated Beers criteria (2015)^27^
Z-drugs	5 (3.5)		
Zolpidem (N05CF02)	5 (3.5)	Nonbenzodiazepines should be avoided, due to possible adverse events and minimal efficacy in treating insomnia. In addition, use of zolpidem can increase emergency department visits and hospitalizations, and motor vehicle crashes; it leads to minimal improvement in sleep latency and duration.	Updated Beers criteria (2015)^27^
Antidepressants	11 (7.7)		
Mirtazapine (N06AX11)	3 (2.1)	These may exacerbate or cause syndromes of inappropriate antidiuretic hormone secretion or hyponatremia. Sodium levels need to be monitored closely when starting use or changing dosages in older adults. They are highly anticholinergic and sedative, and cause orthostatic hypotension.	Updated Beers criteria (2015)^27^
Amitriptyline (N06AA09)	2 (1.4)		Updated Beers criteria (2003 and 2015);^26^^,^^27^ STOPP/START v.2;^28^ French consensus panel^29^
Nortriptyline (N06AA10)	2 (1.4)		Updated Beers criteria (2015)^27^
Clomipramine (N06AA04)	1 (0.7)		Updated Beers criteria (2015)^27^
Paroxetine (N06AB05)	1 (0.7)		Updated Beers criteria (2015)^27^
Fluoxetine (N06AB03)	2 (1.4)	This drug carries a risk of producing excessive stimulation of the central nervous system, sleep disturbances and increased agitation. In addition, there are risks of ataxia, worsening of psychomotor function and syncope. It may exacerbate or cause syndromes of inappropriate secretion of antidiuretic hormone or hyponatremia.	Updated Beers criteria (2003)^26^
Antiepileptics	3 (2.1)		
Phenobarbital (N03AA02)	2 (1.4)	High rate of physical dependence and greater risk of overdose at low dosages.	Updated Beers criteria (2015)^27^
Oxcarbazepine (N03AF02)	1 (0.7)	May exacerbate or cause syndromes of inappropriate antidiuretic hormone secretion or hyponatremia; sodium levels need to be monitored closely when starting use or changing dosages in older adults.	Updated Beers criteria (2015)^27^
Proton pump inhibitors	10 (8.0)		
Omeprazole (A02BC01)	7 (4.9)	Risk of *Clostridium difficile* infection and bone loss and fractures. Avoid scheduled use for > 8 weeks unless for high‐risk patients (e.g. oral corticosteroids or chronic NSAID use) or for erosive esophagitis, Barrett’s esophagitis, pathological hypersecretory condition or demonstrated need for maintenance treatment (e.g. due to failure of drug discontinuation trial or H2 blockers).	Updated Beers criteria (2015)^27^
Pantoprazole (A02BC02)	3 (2.1)		Updated Beers criteria (2015)^27^
Diuretics	6 (4.2)		
Spironolactone (C03DA01)	6 (4.2)	In elderly patients with a creatinine clearance of less than 30 ml/min, serum potassium levels may be increased.	Updated Beers criteria (2015)^27^
Antihypertensives	12 (9.1)		
Immediate-release nifedipine (C08CA05)	7 (4.9)	Potential for hypotension; risk of precipitating myocardial ischemia and constipation.	Updated Beers criteria (2003 and 2015);^26^^,^^27^ French consensus panel^29^
Propranolol (C07AA05)	5 (3.5)	-	Straand criteria (1999)^31^
Doxazosin (C02CA04)	1 (0.7)	Increases risk of orthostatic hypotension or bradycardia.	Updated Beers criteria (2003 and 2015)^26^^,^^27^
Antihyperglycemic	2 (1.4)		
Glibenclamide (A10BB01)	2 (1.4)	All sulfonylureas in general should be avoided among the elderly, because they can cause prolonged hypoglycemia and inappropriate secretion of antidiuretic hormone.	Updated Beers criteria (2015);^27^ STOPP/START v.2^28^
Anti-Parkinsonian	1 (0.7)		
Biperiden (N04AA02)	1 (0.7)	Inappropriate for elderly people with dementia and delirium because it may worsen the cognitive and delirium.	Updated Beers criteria (2015);^27^STOPP/START v.2;^28^ French consensus panel^29^
Antimicrobial	1 (0.7)		
Nitrofurantoin (J01XE01)	1 (0.7)	Potential for renal impairment, pulmonary toxicity, hepatoxicity, peripheral neuropathy or allergic reactions, especially with long‐term use. Bacterial resistance in cases of protracted use can be observed.	Updated Beers criteria (2003 and 2015);^26^^,^^27^ French consensus panel^29^
Antiarrhythmics	1 (0.7)		
Amiodarone (C01BD01)	1 (0.7)	Can be associated with QT interval problems and risk of provoking torsades de pointes. Amiodarone is effective for maintaining sinus rhythm but has greater toxicity than other antiarrhythmics used in atrial fibrillation.	Updated Beers criteria (2003 and 2015)^26^^,^^27^
Total number of PIMs Total number of elderly individuals Average number of PIMs/elderly individual [SD]	159 91 (636%) 186 (0.92)		
Potentially inappropriate interaction (PII)			
Morbidity - drug			
Use of antipsychotics for non-psychotic diagnosis and in dementia	61	The risk/benefit relationship cannot be justified. Use should be avoided, due to adverse events at the central nervous system level.	Updated Beers criteria (2015)^27^
Dementia versus use of benzodiazepines and benzodiazepine receptor agonists	41	Aggravation of cognitive impairment. Possible adverse drug events at central level.	Updated Beers criteria (2015);^27^ French consensus panel^29^
Depression versus long-term use of benzodiazepines	32	May produce or exacerbated depression.	Updated Beers criteria (2003)^26^
Cognitive impairment versus use of tricyclic antidepressants	7	Risk of worsening cognitive impairment.	STOPP/START v.2^28^
Depression versus active metabolites of tricyclic antidepressants	7	May cause anticholinergic side effects.	Canada national consensus panel^30^
Absence of clinical history of coronary and cerebral symptoms and vascular peripheral occlusion; diabetes mellitus; arterial hypertension versus use of acetylsalicylic acid	4		STOPP/START v.2^28^
Dementia versus use of neuroleptics	3	Risk of worsening cognitive impairment.	French consensus panel^29^
Cognitive impairment versus use of barbiturates	2	Concern about effects on the central nervous system.	Updated Beers criteria (2003)^26^
Parkinson’s disease versus use of all antipsychotics except quetiapine	2	Concern due to their antidopaminergic and cholinergic effects.	Updated Beers criteria (2003)^26^^,^^27^
Diabetes mellitus versus use of corticosteroids	1	May worsen diabetes mellitus; serum glucose levels need to be monitored.	Canada national consensus panel^30^
Drug - drug			
≥ 3 CNS-active drugs** in use	17	Increased risk of falls and fractures.	Updated Beers criteria (2015)^27^
Total number of PIIs	177		
Total number of elderly individuals	91 (63.6%)		
Average number of PIIs/elderly individual [SD]	2 [1.42]		

n = number of elderly; SD = standard deviation; ATC = Anatomical Therapeutic Chemical.

*An elderly person can make use of more than one BZD simultaneously; **CNS (central nervous system)-active drugs: antipsychotics, benzodiazepines, nonbenzodiazepines, tricyclic antidepressants, selective serotonin reuptake inhibitors and opioids.

It was observed that 27.3% [39/143] of the elderly people used benzodiazepine medications for insomnia or anxiety, or as a coadjutant for treating depression, and 3.5% [5/143] used zolpidem for sleep disorders. Therefore, around one in four elderly people used BZD after receiving the diagnosis of probable AD.

There were relatives and caregivers who stated that the elderly individuals never used BZD (55), while 16 did not know the history of use. Seventy-one elderly people (49.6%) stated that they had made use of BZD at some time during their lives. Twenty elderly patients had used it before receiving the diagnosis of AD and 19 started to use it after receiving this diagnosis: thus, 39 elderly people were using BZD. The BZDs that these individuals had used most during their lives were clonazepam, bromazepam, diazepam and flunitrazepam (long half-life). The length of use of BZDs ranged from one day to 50 years.

**Figure 2.**
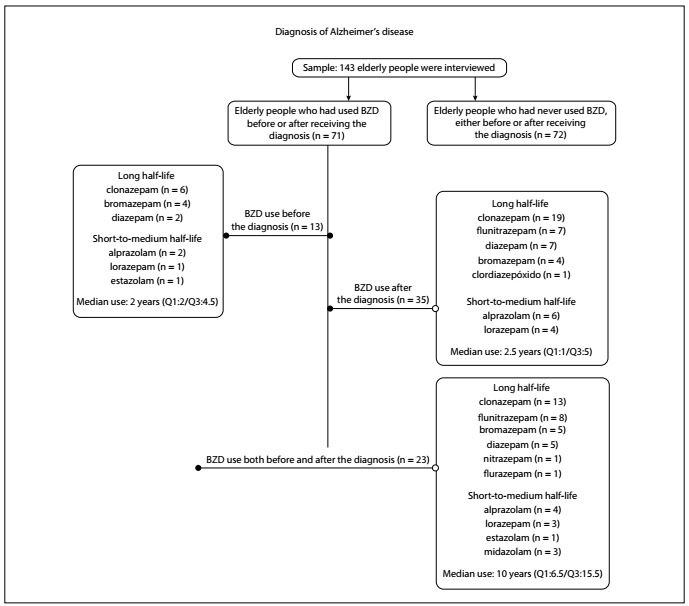
Description of the use of benzodiazepines (BZD) during the patients’ lives, in relation to their diagnoses of Alzheimer’s disease (n = 143), Araraquara, 2017.

Therefore, it was possible to delineate a timeline for the use of benzodiazepines at some point in life, in relation to the diagnosis of probable Alzheimer’s disease, among these elderly people. It was observed that the numbers of elderly patients taking BZD before (36) and after (35) receiving the diagnosis of AD were similar (Figure 2).

Only 9.1% and 1.4% of the elderly patients declared that they were alcohol users and smokers, respectively. However, 24 elderly people were former alcohol users and 49 former smokers.

After multivariate logistic regression, the severity of AD was found not to be a risk factor for the presence of the following drug risk variables: PIM use (P-value: 0.6838), occurrence of PII (P-value: 0.6838), use of polypharmacy (P-value: 0.0781) and occurrence of high-complexity MRCI (P-value: 0.8419).

However, from another perspective, simple logistic regression to assess whether the other variables identified in this study were risk factors for AD severity (such as sociodemographic characteristics or comorbidities, among others) showed that polypharmacy was the only possible risk factor for severity (P-value: 0.0206).

In addition, although comorbidities were not associated with AD severity, it was found that dyslipidemia (P-value: 0.0110), diabetes (P-value: 0.0352), hypertension (P-value: 0.041) and metabolic syndrome (P-value: 0.0376) were associated as risk factors for the presence of at least one of the drug risk variables.

Furthermore, from analysis on all comorbidities according to their ICD-10 classifications, the classes of mental and behavioral disorders (P-value: 0.0109) and circulatory system diseases (P-value: 0.0010) were also found to be associated with the presence of drug risk variables.

## DISCUSSION

Most of the elderly people with AD, in the present study, showed drug risks due to polypharmacy and because they had comorbidities of anxiety, high cholesterol, hypertension, diabetes and metabolic syndrome.

It was observed that the drug therapy for these elderly individuals with AD was of low or medium complexity. We did not find any studies in the literature that identified or discussed the MRCI among elderly people with AD. However, a recent study showed that cognitive impairment was associated with lower MRCI scores than those of other chronic diseases.^32^

The complexity of drug therapy varies according to the morbidity that elderly patients present.^32^ There are correlations not only with the number of drugs in use, but also with other factors such as dosage form and dosage schedule.^24^

On the other hand, although the drug therapy was assessed as presenting low to medium complexity, most of the elderly patients were using five or more drugs. Moreover, simple logistic regression showed that polypharmacy was the only drug risk factor associated with AD severity. Although we are not aware of any studies that have identified an association between polypharmacy and AD severity, there have been some recent studies showing that polypharmacy is a risk factor for dementia.^33^^,^^34^

Other studies have also correlated polypharmacy with prescription of PIMs^1^ and occurrence of PIIs.^3^ Presence of these factors is associated with increased mortality among elderly people.^35^^,^^36^

In the present study, the most commonly used PIM drug classes with PIMs and PIIs were antipsychotics and BZDs. Both of these classes are commonly prescribed for management of behavioral and psychological symptoms of dementia (BPSD).^37^^,^^38^

Even though antipsychotics are frequently prescribed for treating BPSD, this is an off-label use of this drug class, according to the Food and Drug Administration, since these are standard drugs for treating schizophrenia.^39^ Opinions regarding the risk/benefit relationship of this use are divergent. While some studies have shown that antipsychotics are efficacious for improvement of BPSD,^40^ others have shown that their use is associated with a more pronounced cognitive and functional decline and a lack of improvement of BPSD.^41^

The BZD class has mainly been prescribed for management of insomnia and anxiety. In contrast, because sleep disorders are usually related to brain changes arising from AD itself, it is not clear whether the drugs actually used are effective, since the use of these drugs in the context of comorbid neurological disease is also considered to be off-label.^42^^,^^43^

With regard to chronic use of BZD as a risk factor for AD, a recent meta-analysis showed that its use was a risk factor.^44^ From another perspective, a systematic review identified studies in which negative effects from BZD on the cognition of elderly people who already had the diagnosis of AD were reported.^45^

However, we did not find any association between use of BZD and cognitive impairment or progression of AD. This may be explained by the chronic use of BZD before and after the diagnosis of AD was made, the absence of the clinical parameters of MMSE and CDR and the small sample size.

In addition, it was observed that MetS and its risk factors (cholesterol, hypertension and diabetes) and anxiety (a comorbidity that is considered to be a prodromal symptom of AD) were associated with drug risk.

MetS was previously shown to be a risk factor for AD.^46^^,^^47^ Its presence allowed the trajectory of the prodromal stage of AD to become significantly extended to the symptomatic stage.^46^ BPSD have been found to be present both before the diagnosis and during the course of the disease.^37^

Anxiety was an aggravating factor for drug safety among the elderly individuals with AD in the present study and was usually treated with BZD. MetS risk factors were treated with immediate-release nifedipine, propranolol and glibenclamide, and all of these were considered to be PIMs. Nevertheless, these PIMs have been standardized as essential by the WHO and by RENAME-Brazil. However, their respective safer therapeutic equivalents for the elderly, i.e. captopril, losartan and metformin, have also been standardized.

Another important finding from the present study was the absence of determinations of clinical MMSE and CDR parameters among the patients. These findings show that no monitoring of the effectiveness of this drug therapy was being done and, consequently, that there was non-compliance with the guidelines recommended in the PCDTDA/MS.^48^ Therefore, the “minor” complexity of the drug therapy for these elderly individuals with AD did not mean that there were no safety issues or drug-related problems (DRPs) to be identified, prevented, monitored and resolved.

In this context, medication therapy management based on the underlying disease, taking into account the comorbidities and therapeutic experience of the patient and family/caregiver, may be form of pharmaceutical care that would be of interest for promoting drug safety, compliance with drug therapy^49^ and resolution of DRPs.^50^^,^^51^

Thus, important data about the drug safety of patients with Alzheimer’s disease were identified through the present study. Our findings suggest that patients with AD should be regarded in an overall manner: not only managing the number of drugs in use or the complexity of drug therapy, but also taking into account the patient’s needs and comorbidities, along with the experience and expectations of the caregiver/familiar regarding the treatment, and the outcomes when drug risk variables are present.

### Limitations of this study

The main limitation of this study was that the severity of AD was assessed through insertion of memantine, because of absence of the clinical parameters of MMSE and CDR. These clinical parameters are considered to be the gold standard and should be ascertained every six months, as recommended by PCDTDA/MS.

Another limitation was the losses of the present study. However, inclusion in this study of incomplete data obtained through interviews with family members/caregivers who were unaware of the clinical history of the elderly patients would have constituted a form of bias. Nonetheless, this inclusion would have diminished the limitations.

Moreover, the cross-sectional design of this study did not allow us to identify causal associations.

## CONCLUSION

Even though the drug therapy of our elderly patients with AD was of low complexity, the majority of these drugs presented safety risks in relation to the comorbidities of anxiety, cholesterol, hypertension, diabetes and metabolic syndrome. Although we did not identify any evidence in the literature that would correlate polypharmacy with AD severity, our data suggest that this is a possible drug safety risk. Off-label use of PIMs, such as risperidone, quetiapine and benzodiazepines like clonazepam and flunitrazepam, also present a drug safety risk for elderly people with AD.
